# Sensitive Chemical and Biological Sensors Based on Phosphorus Dendrimers

**DOI:** 10.3390/polym17121591

**Published:** 2025-06-06

**Authors:** Anne-Marie Caminade

**Affiliations:** 1 Laboratoire de Chimie de Coordination (LCC-CNRS), 205 Route de Narbonne, 31077 Toulouse CEDEX 4, France; anne-marie.caminade@lcc-toulouse.fr; 2 LCC-CNRS, Université de Toulouse, CNRS, 31013 Toulouse, France

**Keywords:** dendrimer, dendron, chemical sensor, biosensor, poly(phosphorhydrazone) dendrimers

## Abstract

Dendrimers are a special type of ball-shaped hyperbranched polymers consisting of branched monomers organized stepwise around a multifunctional core. They possess many reactive functions, and they are easily accessible as they are located on the surface of the dendrimers. By modifying their terminal functions, it is possible to change the specificities of dendrimers to give them the desired properties. Dendrimers have been used as catalysts, in diverse fields of nanomedicine, and for the elaboration or modification of materials. The internal structure of dendrimers should be carefully chosen depending on the sought-after properties. Poly(phosphorhydrazone) (PPH) dendrimers possess a relatively rigid and hydrophobic internal structure and an easily functionalized surface, which make them appealing in the field of materials. Indeed, they can be used as a matrix, as glue for stabilizing multilayers, or as multifunctional tools. This review describes the use of PPH dendrimers and dendrons (dendritic wedges) for elaborating sensitive chemical, electrochemical, and biological sensors.

## 1. Introduction

**Scheme 1:**
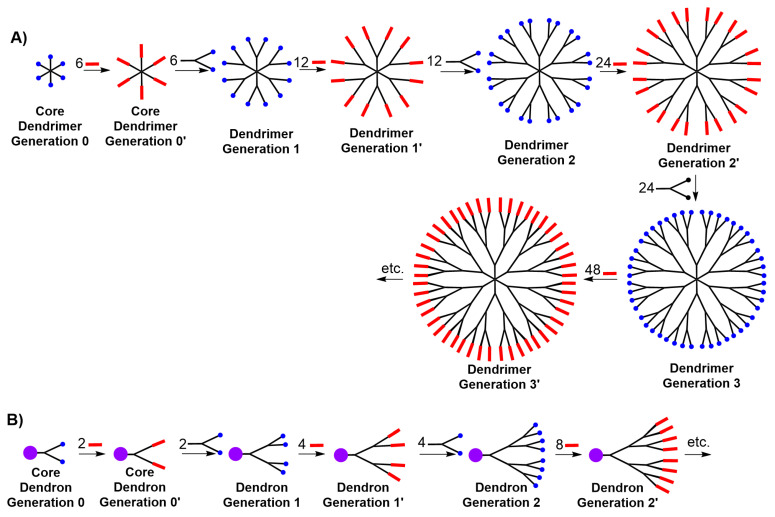
Schematization of the divergent synthesis of dendrimers (**A**) and dendrons (**B**).

Dendrimers [1] are a kind of special type of hyperbranched polymers [2], synthesized step-by-step to ensure a perfectly controlled structure [3]. The synthesis of dendrimers generally requires the repetition of two quantitative reactions, starting from a multifunctional core, and using at least one type of branched monomer, as schematized in Scheme 1. Besides dendrimers, other types of dendritic structures named dendrons (dendritic wedges) [4] have also been synthesized. Dendrons also consist of branches, as dendrimers, but they have one function linked to the core, which is different from the functions on their surface. Dendrons can be synthesized by a divergent method, as dendrimers, or by a convergent method, from the surface to the core.

The divergent method is widely used for the synthesis of dendrimers. It enables an easy functionalization of the surface at each step and the synthesis of large generations, but the presence of defects, especially for high generations, is difficult to avoid due to an increasing number of reactions at each step. The convergent method is mainly used for the synthesis of dendrons. It affords clean products, as only a limited number of reactions are required at each step, but large generations cannot be attained due to an increasing difficulty in accessing the core [5].

The first types of dendrimers were purely organic compounds, with either nitrogen as branching points in the case of PAMAM (poly-amidoamine) [6] or PPI (poly-propyleneimine) [7,8] dendrimers, or carbon in the case of PLys (poly-L-lysine) dendrimers [9]. All these dendrimers have a flexible and rather hydrophilic internal structure. Besides organic dendrimers, inorganic dendrimers are also known [10], having, in particular, phosphorus or silicon as branching elements and a hydrophobic internal structure [11].

**Scheme 2:**
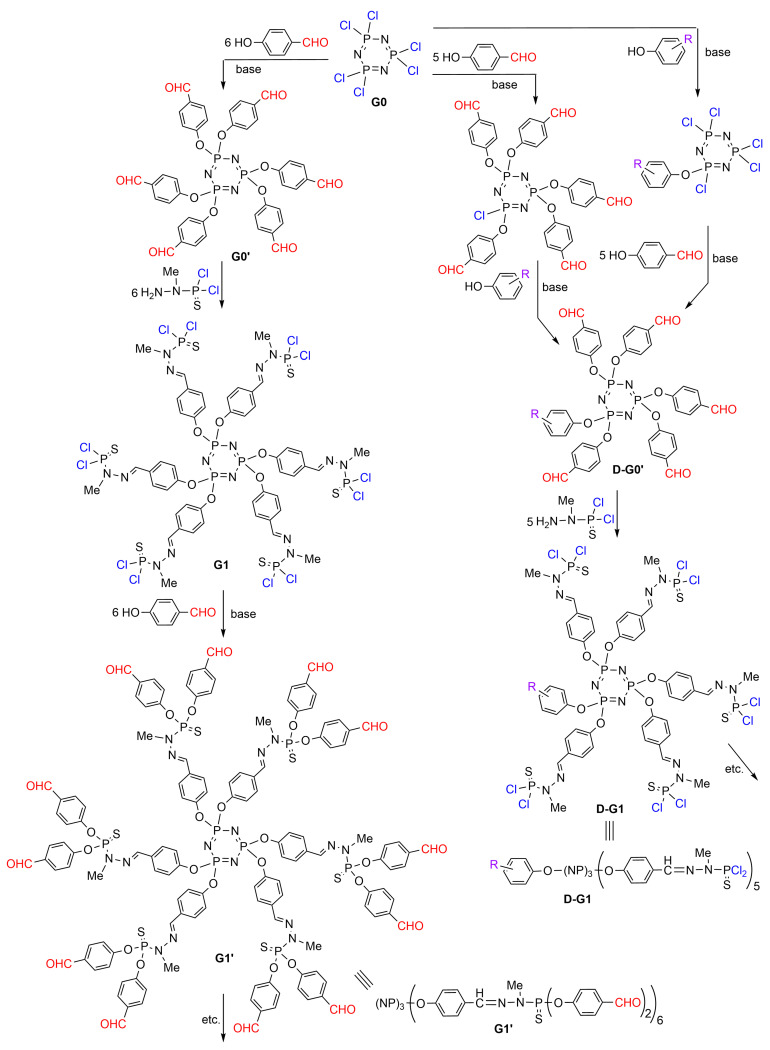
Synthesis of PPH dendrimers and dendrons. Both full structures and the corresponding linear structures are shown for dendrimer **G1’** and dendron **D-G1**.

In the field of phosphorus-containing dendrimers, those based on polyphosphorhydrazone linkages (PPH) are well-known, either built from P(S)Cl_3_ as the core [12] or from cyclotriphosphazene (N=PCl_2_)_3_ as the core [13]. These dendrimers are synthesized by the repetition of two quantitative reactions, which are the substitution of the Cl by hydroxybenzaldehyde and the condensation of the aldehydes with a phosphorhydrazide, as illustrated in Scheme 2 (left part). It becomes more and more difficult to draw the full structure of dendrimers as the generation number increases; thus, another method for drawing dendrimers has been implemented that is a linear way, with parentheses after each level of branching point, as shown at the bottom of Scheme 2. Such a drawing will be used in most figures of this review.

Both reactions are quantitative and afford either aldehyde (family **Gn’**) or P(S)Cl_2_ (family **Gn**) terminal functions, which are particularly suitable for grafting almost any type of functional group to the surface of PPH dendrimers. Substitution reactions with functionalized phenols are the most widely used types of reactions on the P(S)Cl_2_ functions, as they afford more stable dendrimers than those based on reactions with primary amines. Indeed, an increased stability is generally observed for PPH dendrimers functionalized with -P(S)(O-Ar)_2_ groups than with -P(S)(NHR)_2_ groups, towards both moisture and time. Interestingly, it is possible to react specifically a single Cl among two to afford specifically difunctional dendrimers [14]. It was recently shown that precise difunctionalization afforded more efficient catalysts than random difunctionalization [15].

The shape and properties of these PPH dendrimers depend in part on the dendrimer generation. Their number of terminal functions is multiplied by two at each generation, as is, approximately, their molecular weight. Their structure obtained by molecular dynamics looks like a cauliflower for the first generation [16], a flattened ball for the intermediate third generation [17], or balls of increasing size, as shown by electron microscopy, for generations above 3 [18]. Such structural modifications make generation 4 PPH dendrimers the most attractive, as they are ball-shaped and obtained after fewer synthetic steps than higher generations.

The use of hexachlorocyclotriphosphazene as the core of dendrimers is particularly interesting, as it enables the specific grafting of either five functions or a single function [19,20] to afford dendrons, as illustrated in Scheme 2 (right part). The growth of the dendrons is then carried out using the same reactions as for the PPH dendrimers to afford dendrons having either aldehyde (family **D-Gn’**) or P(S)Cl_2_ (family **D-Gn**) terminal functions. As in the case of dendrimers, dendrons can also be drawn in a linear structure with parentheses after each level of branching points, as shown in the lower part of Scheme 2.

The terminal functions are suitable for performing a versatile reactivity, as for dendrimers. Depending on the nature of the function linked to the core (the R group), such dendrons can be grafted together to obtain Janus dendrimers [21] or grafted to materials [22], or they can form micelles in water that are suitable for biological experiments [23].

PPH dendrimers and dendrons possess three distinct advantages over all the other types of dendrimers [24], as follows: (i) a hydrophobic internal structure, (ii) relatively rigid branches due to the flat O-C_6_CH_4_-CH=N-N(Me)P(S) linkage, as shown by the crystal structure of several first generations [25,26,27], and (iii) their characterization and the easy detection of structural defects thanks to ^31^P NMR [28], even for very high generations [27]. Additionally, both the aldehyde and P(S)Cl_2_ terminal functions available at each synthetic step are among the most reactive types of functions and are suitable for grafting almost any type of functional group to give the dendrimers the desired properties. All these advantages make PPH dendrimers potentially useful in the field of sensors.

This review will present the contribution of such PPH dendrimers and dendrons to the elaboration of chemical and biological sensors. Two old reviews have gathered the early history of the field for DNA microarrays [29] and for sensors [30].

Sensors are of widespread interest in chemistry [31,32,33], electrochemistry [34,35], and biology [36,37,38]. Sensors are frequently based on polymeric materials [39,40], but few are based on dendrimers, and the corresponding reviews are generally highly specialized [41,42,43], particularly regarding dendrimer type, such as ferrocenes [44,45] or PAMAM [46].

## 2. Phosphorus Dendrimers as Chemical Sensors

**Figure 1:**
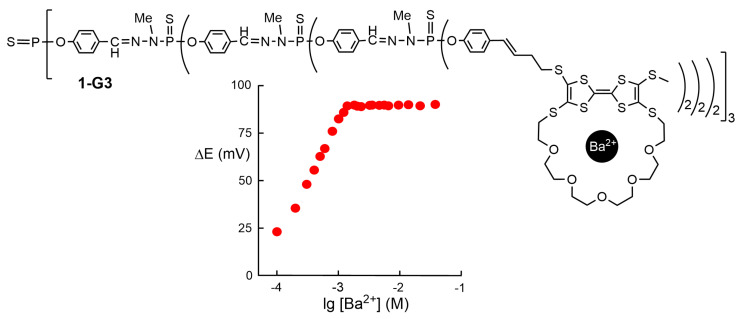
PPH dendrimer **1-G3** functionalized with 24 TTF/crown ether macrocycles and used as electrochemical sensor for Ba^2+^ [47].

The very first example of a phosphorus dendrimer used as a sensor concerned a third-generation PPH dendrimer built from a trifunctional core, and it was equipped on the surface with 24 tetrathiafulvalene (TTF)/crown ether (CE) macrocycles (**1-G3**, Figure 1). These dendrimers were built up to the fifth generation (96 TTF/CE macrocycles). TTF is a well-known redox-active subunit with three different stable redox stages (neutral, radical cation, and bication). Such a property allows, in particular, the electrochemical release of metallic cations to be controlled. The electrodeposition of the dendrimers was performed by recurrent potential scans on a Pt working electrode, and also on gold or glassy-carbon electrodes. The macrocycle shown in Figure 1 is suitable for the encapsulation of barium cations (Ba^2+^). Adding a solution containing increasing concentrations of Ba^2+^ cations (from 1 10^−4^ to 8 10^−4^) to the dendrimer **1-G3** deposited onto a Pt electrode induced, as an electrochemical response, a progressive positive shift of the first TTF wave (E), followed by a constant E value when the saturation point is attained (graph in Figure 1) [47].

**Figure 2:**
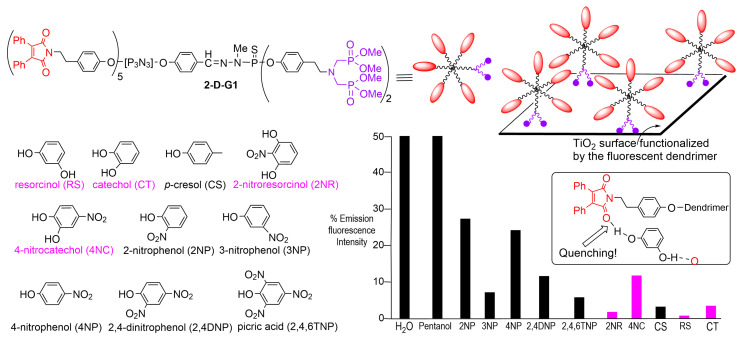
Fluorescent dendron **2-D-G1** grafted to a TiO_2_ film and used for the detection of nitrophenols; efficiency of the fluorescence quenching, depending on the nitrophenol quencher used [48].

The second example of a chemical sensor was obtained with the small dendron **2-D-G1**, functionalized with five maleimide chromophores on one side and two azabisphosphonate groups on the other side (Figure 2). The azabisphosphonates were suitable for the covalent grafting of dendron **2-D-G1** to a titanium oxide (TiO_2_) film surface by dip-coating. The films were characterized, in particular, by steady-state fluorescence, with a maximum emission at λ = 515 nm. Quenching experiments were carried out by dipping the film into stock solutions of different hazardous phenolic compounds in chloroform, listed in Figure 2, and also water and pentanol. The quenching is attributed to the formation of hydrogen bonds between OH and the carbonyl groups of the dendrons. Quenching was more efficient with phenols than with alcohols or water, and aryl groups bearing two OH groups (compounds in pink color in Figure 2) were among the most efficiently detected compounds, especially those from resorcinol and 2-nitroresorcinol, presumably thanks to the ability of a single diol molecule to quench two maleimide functions. Attempts to recover the fluorescence emission were carried out in the case of the quenching by 2-nitroresorcinol by soaking the device in basic solutions from pH 7.5 to 10. The fluorescence recovery was identical to that measured in water when pH 10 was reached [48].

**Figure 3:**
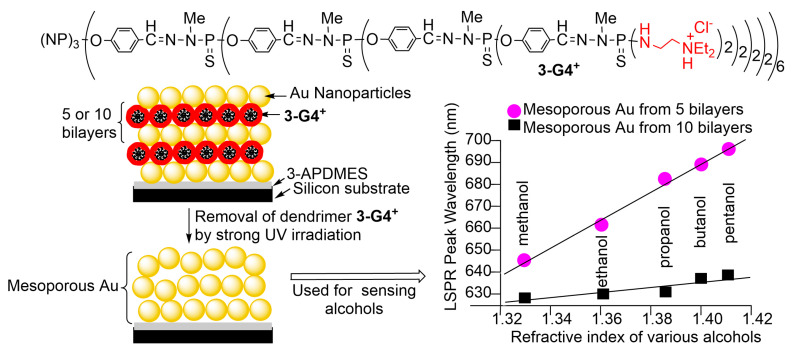
LbL experiments with negatively charged Au_NPs_ and dendrimer **3-G4^+^**, then removal of the dendrimer and use of the Au_NPs_ device for sensing alcohols, as measured by LSPR [55].

A series of alcohols was also detected in another experiment, starting with a generation four dendrimer bearing 96 ammoniums as terminal functions (compound **3-G4^+^** in Figure 3). Anionic gold nanoparticles (AuNPs with diameters of either ca 3 nm or ca 16 nm) were first deposited onto 3-(diethoxymethyl-silyl)propylamine (3-APDMES)-coated silicon substrates, followed by a layer of dendrimer **3-G4^+^**. Multilayer thin films were obtained using a layer-by-layer (LbL) assembly, driven by electrostatic interactions. The layer-by-layer (LbL) deposition of alternating positively and negatively charged entities, in particular polymers [49], has generated a wide interest, with many applications for the generation of nanocomposite materials [50]. The dendrimers enable a more precise organization of the multilayer than polymers do. Five or ten bilayers were deposited in this way. Then, the dendrimers were removed using strong UV irradiation, as it was previously demonstrated that PPH dendrimers are largely fragmented in MALDI-tof mass spectrometry experiments using a UV laser [51]. The clean destruction and elimination of the PPH dendrimers by UV irradiation allows for preserving the organization of the gold layers, which is a unique advantage of PPH dendrimers among all types of dendrimers. The resulting Au_NPs_ multilayers were used for the detection of five alcohols with different refractive indexes (Figure 3, right graph). Measurements were carried out by immersion of the device in the alcohol, and then they were analyzed by LSPR (localized Surface Plasmon Resonance), a sensitive sensor method [52,53,54], which displayed a moderate red shift of the peak maximum of Au multilayer films with increasing refractive index values of the solvents. The effect was more pronounced in the case of the device obtained from ten than from five bilayers (graph in Figure 3) [55].

**Figure 4:**
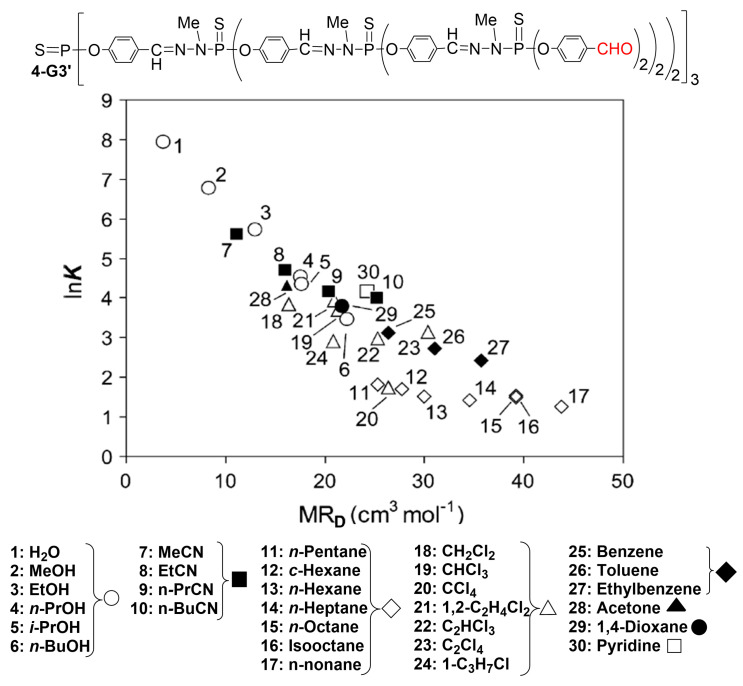
Thick layers of dendrimers of type **4-Gn’** (n = 1–4, 9) used for sensing 30 vapor solvents using a quartz microbalance sensor. Graph of guest partition coefficients ln *K* and molar refraction of the guests *MR_D_* obtained with dendrimer **4-G3’** [56].

Other experiments were carried out with thick layers of dendrimers deposited by solution drop and drying on a quartz microbalance sensor, and they were used for their sorption capacities for 30 volatile guests (solvent vapors). Dendrimers with aldehyde terminal functions and built from a trifunctional core were tested, from generations 1 to 4 and also generation 9 [18,25]. The correlation between ln *K* (derived from the modification of the quartz microbalance vibration) and *MR_D_* (guest molar refraction), shown in Figure 4 for dendrimer **4-G3’**, indicates that each solvent has a unique characteristic and that a relative correlation can be found in each family of solvents. Analogous correlations were obtained with all dendrimers tested, but with different selectivity. This selectivity is lower than that of calixarenes but is largely higher than that of polymers. The best sensing results deduced from the variation of the quartz frequency (ΔF in Hz) were observed, in many cases, with generation 4 dendrimer **4-G4’**, in particular for all nitriles (from Me-CN to n-BuCN), and for CH_2_Cl_2_, 1,2-C_2_H_4_Cl_2_, C_2_HCl_3_, 1-C_3_H_7_Cl, benzene, toluene, ethyl benzene, acetone, 1,4-dioxane, pyridine, or water. The generation 3 dendrimer **4-G3’** was also found very efficient, in particular for sensing all alkanes (*i*-PrOH, *n*-pentane, *c*-hexane, *n*-hexane, *n*-heptane, *n*-octane, isooctane, *n*-nonane), CHCl_3_, or C_2_Cl_4_. The generation 1 dendrimer **4-G1’** was particularly useful for sensing alcohols (methanol, ethanol, *n*-PrOH, *n*-BuOH) or CCl_4_, whereas the sensing capacities of generation 2 (**4-G2’**) and generation 9 (**4-G9’**) were lower in all cases than that of generations 3 and 4 and even 1 [56].

## 3. Phosphorus Dendrimers as Biological Sensors

**Scheme 3:**
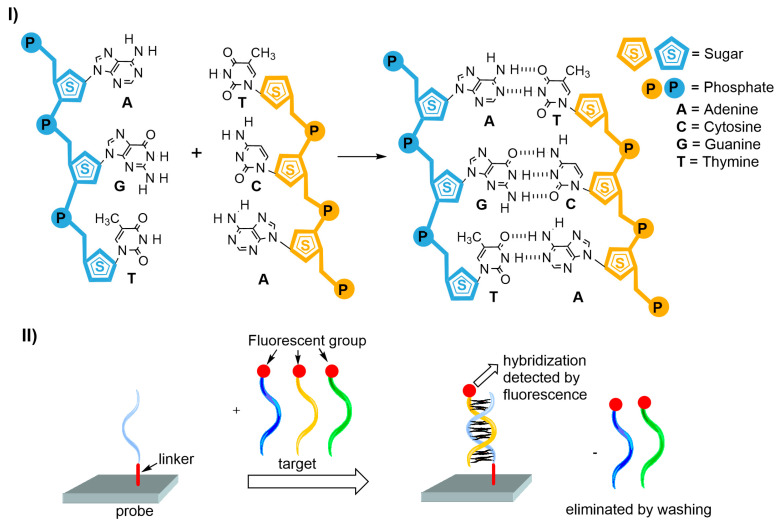
(**I**) Principle of hybridization between complementary bases. (**II**) Principle of a biochip, and detection of the hybridization by fluorescence.

All biological sensors that will be described in this part of the review use the association between complementary bases of two single-stranded DNA fragments or oligonucleotides, which is named hybridization. Hydrogen bonds that occur between adenine (A) and thymine (T), and between cytosine (C) and guanine (G), induce such hybridization. This concept is illustrated in part I of Scheme 3. Biological sensors are obtained by linking one oligonucleotide of known structure (the probe) to a surface (generally glass), then adding a water solution of fluorescently labelled oligonucleotides. Only the complementary oligonucleotide will hybridize, and the hybridization is detected by fluorescence (part II of Scheme 3) [29]. The structure of the target oligonucleotide is deduced from that of the probe, using the A-T and C-G correlation. The nature of the linker between the surface and the probe is of utmost importance for the sensitivity of these biochips, as will be illustrated in the next paragraphs.

### 3.1. DNA Microarrays Based on a Single Layer of Dendrimers

**Figure 5:**
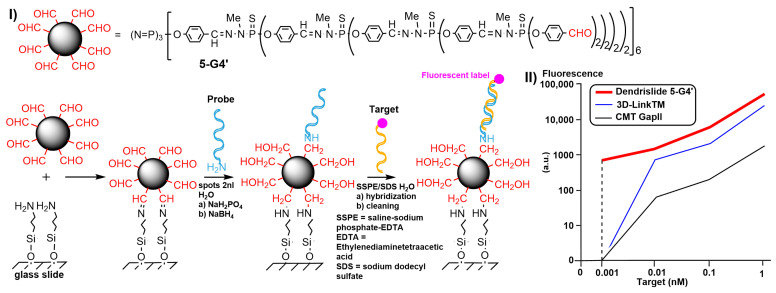
(**I**) Elaboration and use of dendrislides. (**II**) Efficiency of the dendrislides compared to other slides. Only the dendrislides are efficient at the concentration of 1 pM (10^−12^ M) in target [57].

Generations one to seven of PPH dendrimers having from 12 to 768 aldehyde terminal functions (family **5-Gn’**, n = 1 to 7) were covalently grafted to amino-silanized glass slides, affording full coverage of the glass slide. A 35mer DNA probe was spotted on the dendrimer layer, which was then hybridized with a 15mer Cy5-labelled complementary strand (Figure 5, part I). The signal-to-background ratio of the slides increased using dendrimers from generation 0 to generation 4 and remained constant from generation 4 to generation 7. Thus, all the other experiments were carried out with dendrimer **5-G4’** to produce dendrislides, as it provided the best trade-off between efficiency and synthetic time and effort. At a very low concentration in the target, 1 pM (10^−12^ M), only the dendrislides were able to detect the hybridization, compared to 12 different other types of slides, as shown in Figure 5 part II (only results from two slides, the most efficient and the least efficient, are shown) [57].

It was shown that these dendrislides can be reused using a stripping procedure consisting of the incubation of the used dendrislides in a stripping buffer at 95 °C for 5 min. Hybridization/stripping cycles were carried out 10 times and afforded the same fluorescence intensity at run 10 as at run 1, demonstrating the robustness of these slides. Furthermore, a single mutation in the middle of the target oligonucleotide prevents hybridization and thus detection, emphasizing another type of sensitivity of these dendrislides [58].

**Figure 6:**
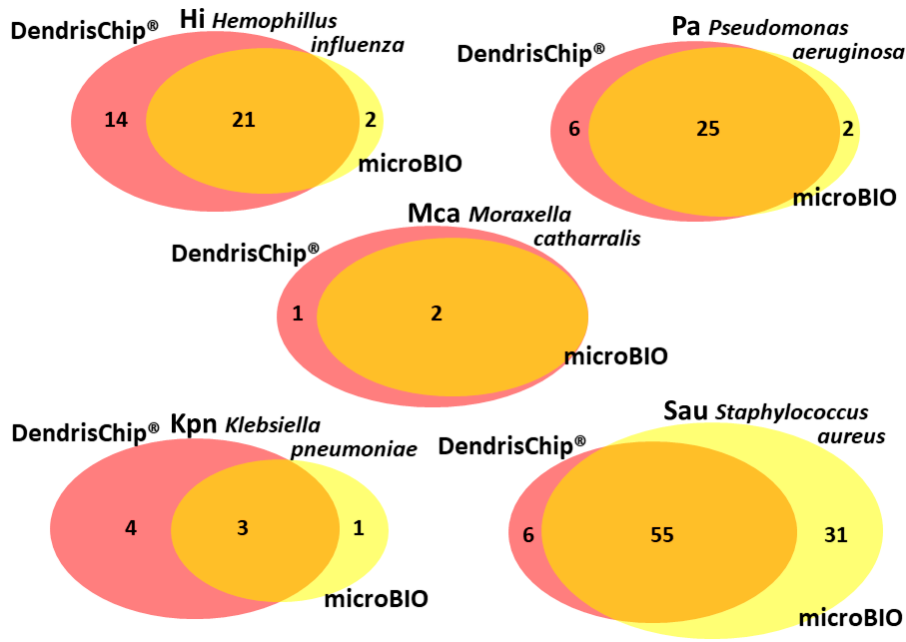
Comparison of positive identification results for five pathogens between the DendrisChips^®^ technology and the conventional microbiology culture (microBIO). Results from 238 clinical samples. Caution: In the case of Sau, only 10 out of the 31 samples positively identified by the microbiological culture were confirmed by PCR assays [60].

In view of all these properties, a start-up named Dendris^TM^ was created to develop these dendrislides, named DendrisChips^®^. These slides enable the spotting of several probes in precise areas, allowing multiplexing, that is, the detection of multiple targets in a single experiment [59]. The first targeted diseases were respiratory infectious diseases, in particular those caused by bacteria. The DendrisChips^®^ were able to detect and discriminate simultaneously in a single experiment 11 bacteria implicated in respiratory tract infections. A diagnostic result is delivered in about 4 h. A large test was carried out with 238 clinical samples, which were analyzed both by standard microbiological cultures and the DendrisChips^®^. Figure 6 displays the comparison of both methods for the detection of five pathogens. In four cases among five, the DendrisChips^®^ have a better sensitivity than culture methods. The large difference observed in the case of *Staphylcoccus aureus* incited the authors to use a specific PCR assay for this pathogen. Only 10 out of the 31 samples positively identified as *Staphylococcus aureus* by the microbiological culture were confirmed, whereas all those identified by the DendrisChip^®^ were confirmed [60].

**Figure 7:**
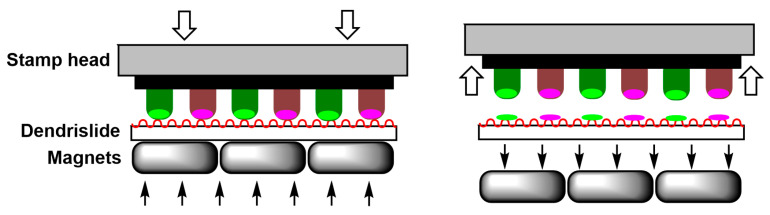
Principle of printing oligonucleotides by soft micrography on a dendrislide [61]. The arrows indicate the movement of stamp head and magnets during the printing process.

The slides functionalized by the **5-G4’** dendrimers have been used for other sensing experiments. In a first example, the oligonucleotides were not spotted but printed by soft micrography using an automated microcontact printing device bearing 64 individual pillars, each mounted with 50 circular micropatterns (spots) (Figure 7). The resulting low-density microarrays showed an excellent hybridization response with complementary sequences at unusually low probe and target concentrations. The actual probe density immobilized by this method was at least 10-fold lower than with the conventional mechanical spotting but afforded a comparable hybridization response between spotted and printed oligoarrays with a 1 nM complementary target [61].

**Figure 8:**
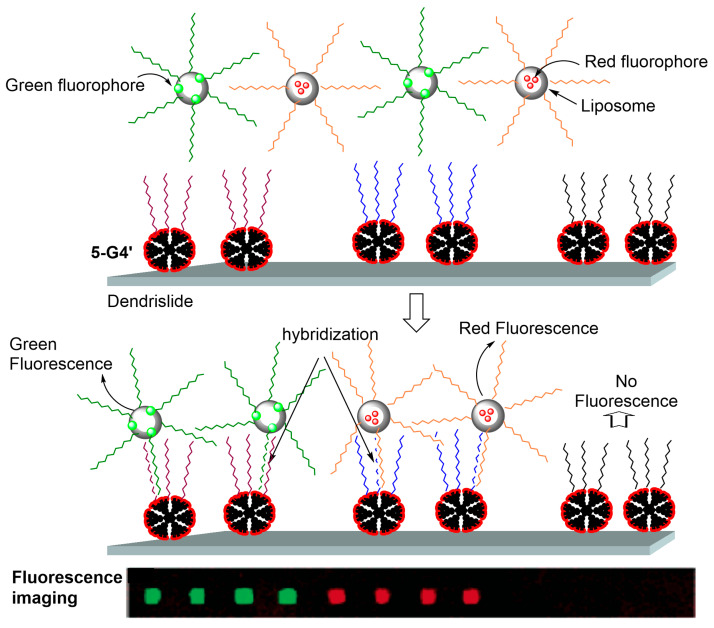
Specific liposome attachment on a dendrislide via oligonucleotide hybridization, and detection by fluorescence [62].

The dendrislides could be converted to liposome arrays by grafting on liposomes oligonucleotides complementary to oligonucleotides bound to the dendrimers. The detection of hybridization was carried out with liposomes encapsulating fluorescent dyes inside the liposomes (Cy-5 red dye), or a fluorescent (green rhodamine) lipid was used for labeling the liposome membrane. A very high specificity of hybridization and detection by fluorescence was observed, with the same sensitivity as that observed in the association of the dendrislides with simple oligonucleotides (Figure 8) [62].

**Figure 9:**
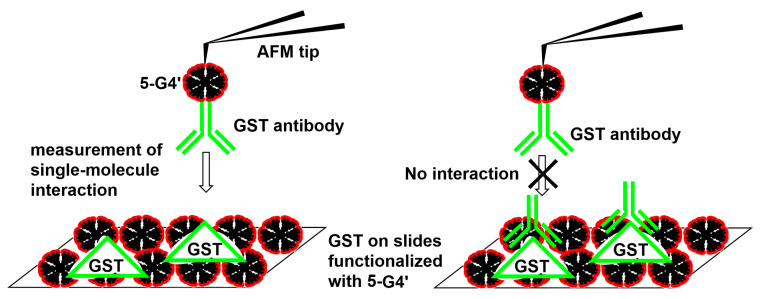
Single-molecule interaction with antibody–dendritip; blocking of specific interactions [63].

In another experiment, Glutathione-S-transferase (GST) was spotted on a dendrislide, and an atomic force microscopy (AFM) tip was functionalized with a **5-G4’** dendrimer supporting a GST antibody. Single-molecule force interactions between GST and GST antibody were probed by AFM spectroscopy and were measured to be 67 ± 11 pN. Blocking tests performed by depositing the GST antibody on GST resulted in the loss of interactions with the GST antibody on the tip (Figure 9) [63].

**Figure 10:**
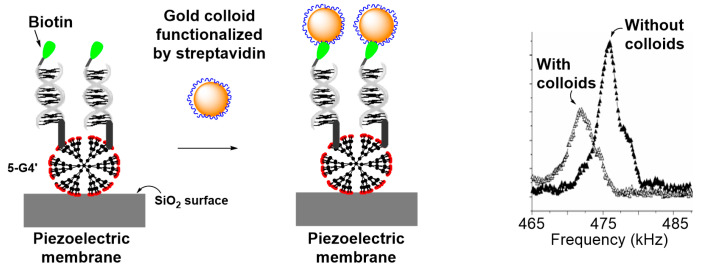
Streptavidin-conjugated gold nanoparticles trapped by biotinylated piezoelectric membrane, and modification of the resonating frequency of the device [64].

Dendrimer **5-G4’** was also grafted to a SiO_2_ surface linked to a resonating piezoelectric membrane. Such a surface was then functionalized with oligonucleotides, and then it was hybridized with a complementary oligonucleotide bearing biotin. Such a device was used for sensing the capture of streptavidin-conjugated gold nanoparticles. A 3.9 kHz shift of the resonant frequency of the resonating piezoelectric membrane was recorded before and after the adsorption of the streptavidin-conjugated gold nanoparticles (Figure 10) [64].

**Figure 11:**
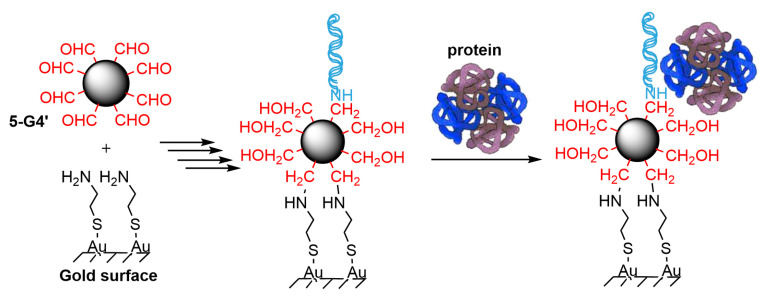
Detection of protein–DNA interactions by Surface Plasmon Resonance imaging [65].

The method for functionalizing glass slides shown in Figure 5 was also applied for the functionalization of gold surfaces. In that case, the gold surface was first reacted with cystamine (H_2_N(CH_2_)_2_S-S(CH_2_)_2_NH_2_), then with the **5-G4’** dendrimer, and then with double-stranded DNA (Figure 11). This device was then applied for determining the interaction of DNA with proteins using Surface Plasmon Resonance imaging (SPRi) to follow the interactions in real time. The presence of the dendrimers significantly improved the selectivity of the detection of protein–DNA interactions by reducing the background signal. Such improvement allowed for discriminating between protein and DNA interactions of different strengths, even in the case of only slightly different affinities [65].

### 3.2. Biosensors Based on Multilayers of Dendrimers

**Figure 12:**
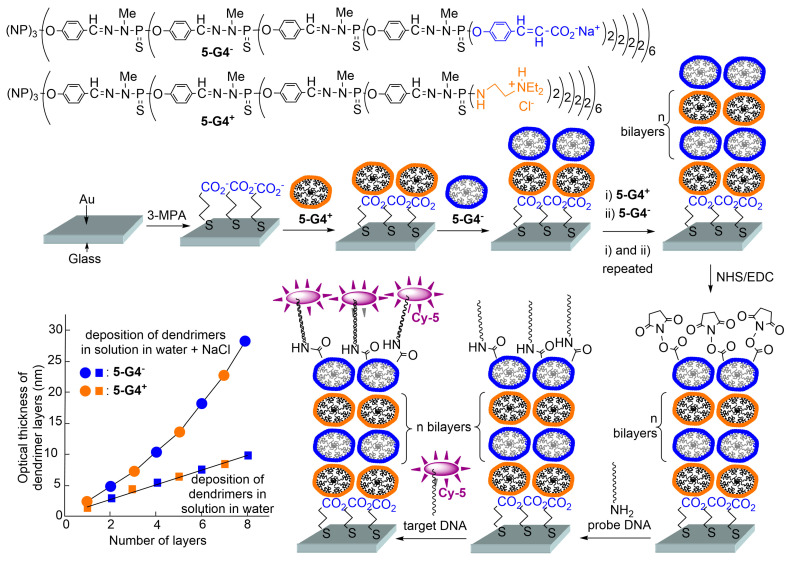
Layer-by-layer deposition of dendrimers **5-G4^+^** and **5-G4^−^**, followed by covalent grafting of DNA probe and hybridization with fluorescent complementary oligonucleotide. Measurement of the thickness of the layers (graph on the left) [66].

The LbL concept shown in Figure 3 has also been applied to positively (**5-G4^+^**) and negatively (**5-G4^−^**) charged generation 4 PPH dendrimers with either tertiary ammoniums or carboxylate salts as terminal functions, respectively. These dendrimers were deposited LbL on a glass surface coated with a thin layer of gold functionalized with 3-mercaptopropionic acid (3-MPA). The dendrimers were deposited either in solution in water or in solution in water + NaCl to increase the thickness of the layers. The growth of the layers was monitored by SPR spectroscopy (graph in Figure 12). Single-stranded oligonucleotides were covalently grafted onto the upper layer of negatively charged dendrimers, and then hybridization was carried out with complementary oligonucleotides labelled with the Cy-5 dye (Figure 12). The limit for the detection of hybridization by fluorescence was measured at 50 pM in the case of a single bilayer of dendrimers and at 30 pM (30 10^−12^ M) in the case of four bilayers of dendrimers. This difference was attributed to the diffusion of probe DNA into the multilayer system, increasing the amount of probe DNA available for the target DNA [66].

**Figure 13:**
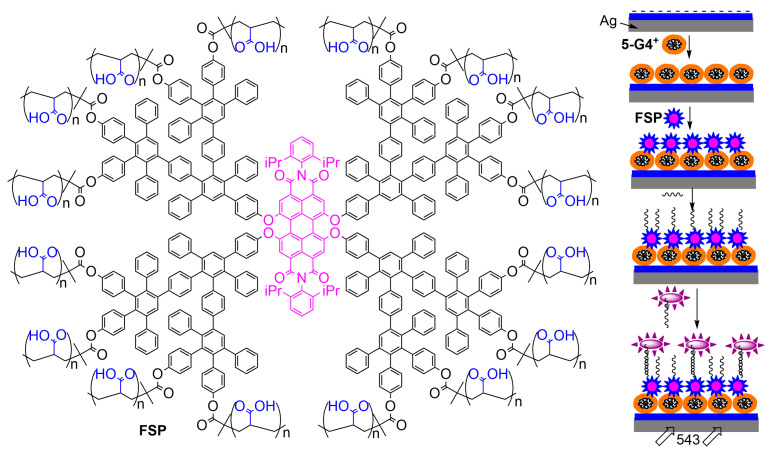
Structure of FSP and principle of the elaboration of an ultrasensitive sensor (10^−18^ M) [67].

A related experiment was carried out with dendrimer **5-G4^+^** and a perylene diimide-labelled star polymer (FSP), both deposited LbL on a negatively charged silver substrate. Dendrimer **5-G4^+^** acted as a matrix, whereas the FSP was selected as a fluorescent donor molecule of high quantum yield. Single-stranded oligonucleotides were covalently grafted onto the FSP layer, and hybridization with complementary fluorescent (Cy5) oligonucleotide (Figure 13) was monitored by the Surface Plasmon Enhanced Fluorescence Spectroscopy (SPFS) technique under excitation at 543 nm. The detected fluorescence signal of Cy5 was mainly due to an efficient excitation energy transfer from FSP to Cy5. Such a method allowed the ultrasensitive and selective detection of DNA targets in relation to enhanced optical fields associated with energy transfer. An increased fluorescence/background ratio was measured even when the concentration of complementary oligonucleotide was down to 10^−18^ M (1 aM), displaying ultrasensitive detection [67].

**Figure 14:**
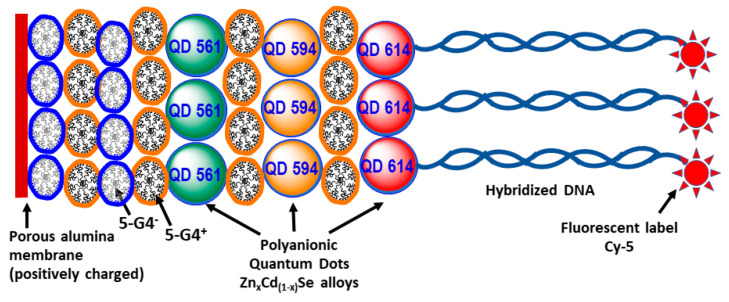
Sensitive graded-bandgap sensor based on dendrimers **5-G4^+^** and **5-G4^−^**, and QDs [71].

The LbL process with dendrimers **5-G4^+^** and **5-G4^−^** was also carried out inside cylindrical nanopores of anodic aluminum oxide membranes of 400 nm in diameter to afford nanotubes of dendrimers after the removal of the alumina template [68]. The same LbL process was also carried out with membranes with pore diameters between 30 and 116 nm and by varying the ionic strength. Deposition within the nanopores can become inhibited, even if the pore diameter is much larger than the diameter of the dendrimers (diameter of about 7 nm [18]), particularly at high ionic strength [69]. Sensors were produced within these nanotubes by replacing some layers of negatively charged dendrimers with layers of negatively charged quantum dots (QDs) based on Zn_x_Cd_(1−x)_Se alloys, with emissions at 561, 594, or 614 nm and organized to afford a graded bandgap [70]. Such a device was used as a biosensor by grafting a single-stranded oligonucleotide on the upper layer of QD^614^, followed by hybridization with a complementary Cy-5-labelled oligonucleotide (Figure 14). Efficient fluorescence resonance energy transfer (FRET) along the bandgap gradient enabled the sensitive detection of hybridization by SPR (Surface Plasmon Resonance) and SPFS spectroscopy [71].

**Figure 15:**
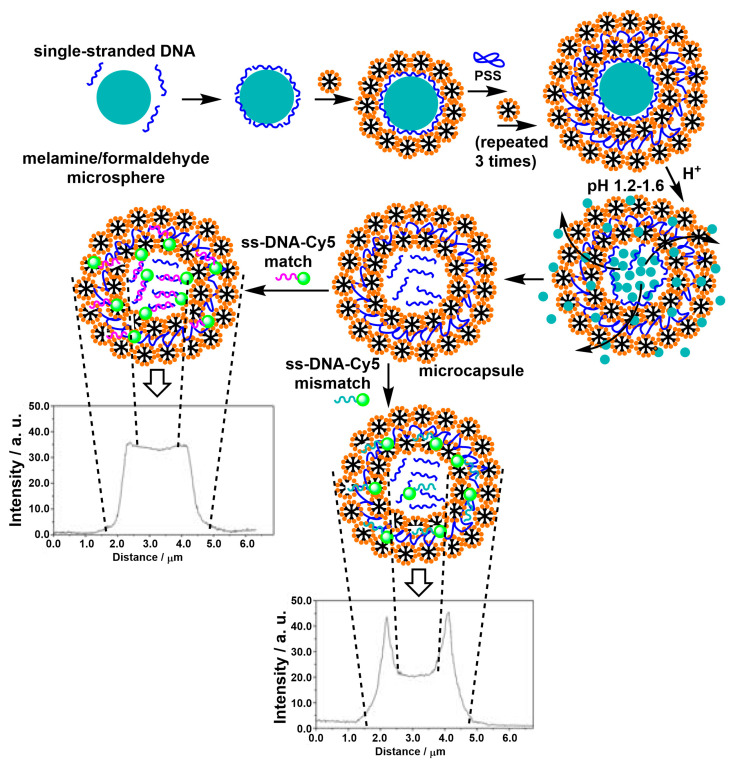
Synthesis of microcapsules and their use as sensors for DNA hybridization. Fluorescence profile in case of match (**left**) and mismatch (**right**). Warning: The size of the microsphere, and thus of the interior of the microcapsule, is larger than the thickness of the multilayer constituting the wall, contrary to the drawings [74,75].

Besides nanotubes made of dendrimers, microcapsules were also obtained by the LbL process by alternating either poly-(allylamine hydrochloride) (PAH) and negatively charged dendrimer **5-G4^−^** or poly(styrenesulfonate) (PSS) and positively charged dendrimer **5-G4^+^** [72], or even DNA and dendrimer **5-G4^+^** [73], onto melamine/formaldehyde microspheres (diameter 7 μm). The microspheres were then destroyed at acidic pHs, and the residues were eliminated through the multilayers, generating microcapsules. To obtain sensors, such a process was applied to single-stranded DNA (ss-DNA), which was deposited first, then dendrimer **5-G4^+^** and PSS were repeatedly deposited (4 times). The elimination of the microsphere template at acidic pHs liberated ss-DNA inside the generated microspheres. Other ss-DNAs functionalized with Cy-5 were then added. In the case of complementarity between the fluorescently labelled ss-DNA and the ss-DNA inside the microcapsule, fluorescence was largely detected inside the microcapsule. In the case of mismatch, fluorescence was detected essentially in the multilayers constituting the wall of the capsule (Figure 15) [74,75].

In all these cases of the LbL elaboration of multilayers, the dendrimers act as a matrix or glue to organize and stabilize these multilayers.

## 4. Conclusions

As illustrated in this review, PPH dendrimers are particularly suitable for the elaboration of chemical, electrochemical, and, above all, biological sensors. Diverse generations were used, from generation 1 to generation 7, but most experiments were carried out with generation 4, which generally afforded the best choice between efficiency and synthetic time and effort. In most cases, the detection is observed by fluorescence, which proved to be highly sensitive, enabling detection at 10^−12^ M for commercial devices [57,60] and even at the very low value of 10^−18^ M for research devices [67]. This approach is linked to the exponential demand for new non-invasive in vitro diagnostic techniques. In the search for more informative and precise methods providing more data from a single biological sample at a fast and cost-effective value, dendrimers can provide smart solutions. A large cooperation between biologists, chemists, computer scientists, engineers, and, above all, industries is needed for the development of such devices for the benefit of mankind.

## Data Availability

No new data were created.
